# Administration of B7-H3 targeted chimeric antigen receptor-T cells induce regression of glioblastoma

**DOI:** 10.1038/s41392-021-00505-7

**Published:** 2021-03-26

**Authors:** Xin Tang, Yuelong Wang, Jianhan Huang, Zongliang Zhang, Fujun Liu, Jianguo Xu, Gang Guo, Wei Wang, Aiping Tong, Liangxue Zhou

**Affiliations:** 1 Department of Neurosurgery, West China Hospital, West China Medical School, Sichuan University, Chengdu, Sichuan Province China; 2 State Key Laboratory of Biotherapy, West China Hospital, West China Medical School, Sichuan University, Chengdu, Sichuan Province China

**Keywords:** Drug development, Immunotherapy

**Dear Editor**,

Nowadays, glioblastoma (GBM) was the most common and lethal form of primary intracranial tumor. Despite standard-of-care therapy, GBM still exhibited a poor prognosis with 5-years survival rate less than 5%. Recent years, adoptive CAR-T therapy came to be a novel immunotherapy in treating malignant tumors. Great progress has been made by CD19 targeted CAR-T cells against refractory B cell cancers. Recent studies also reported about the clinical potential of CAR-T therapy targeting IL13Ra2 and EGFRvIII in treating GBM.^1,2^ However, limited numbers of therapeutic targets in GBM may preclude it from progress and being popularized.

B7-H3 (CD276) has been found to be overexpressed by many tumors and tumor-infiltrating dendritic cell.^3^ Our previous studies suggested the potent anti-tumor effect of B7-H3 targeted CAR-T cells against GBM in preclinical models.^4^ Here we presented our clinical experience with one patient to evaluate the therapeutic potential of B7-H3 targeted CAR T-cell therapy in treating recurrent GBM.

In this case, a 56-year-old woman presented with recurrent GBM in the left frontal and parietal lobe (Supplementary Fig. 1). The patient has received twice craniotomy and standard-of-care with chemoradiation in the last 2 years. Pathologic study of tumor resection showed 50% expression of Ki67 and a high but heterogeneous B7-H3 expression, with a histochemistry score evaluated as 110 (0–300) (Supplementary Fig. 2a). Flow cytometry assay of tumor primary cells also confirmed the high B7-H3 expression (Supplementary Fig. 2b).

In the preclinical study, we identified the specific tumor-lysis ability of autologous B7-H3 targeted CAR-T cells. The structure of B7-H3-targeted CAR was shown in Supplementary Fig. 2c. Flow cytometry results indicated CAR-T cells displayed memory T cell markers (CD45RO and CD62L), and had relative low levels of or were negative for effector T-cell markers (CD69 and CD25) and PD-1/Tim-3 (Supplementary Fig. 2d). In a real-time monitoring of cytotoxicity assay, B7-H3 targeted CAR-T cells induced specific anti-tumor effect in tumor primary cells (Supplementary Fig. 2e). Enzyme-linked immunosorbent assay (ELISA) results also indicated an activation effect of the CAR-T cells when cocultured with tumor primary cells (Supplementary Fig. 2f).

Three weeks after the craniotomy, tumor recurrence was found in the surgical resection site by magnetic resonance imaging (MRI). The patient received weekly intracavitary infusions of B7-H3 targeted CAR-T cells. The first two round infusion was following a dose-escalating principle (Fig. 1a). The CAR-T cells was delivered by an Ommaya device (Supplementary Fig. 1b). After the first-round infusion, we observed a dramatic reduction of recurrent tumor by MRI. Remarkably, the enhanced part of the recurrent tumor was significantly reduced, compared to the signal before infusion (Fig. 1b). The clinical response was sustained for about 50 days after the initiation of CAR-T cells infusion. Unfortunately, this patient appeared in drowsiness and altered consciousness in cycle 6 and 7 and MRI revealed tumor recurrence. Finally, the patient dropped out of the clinical study after the 7 cycles infusion.

**Fig. 1:**
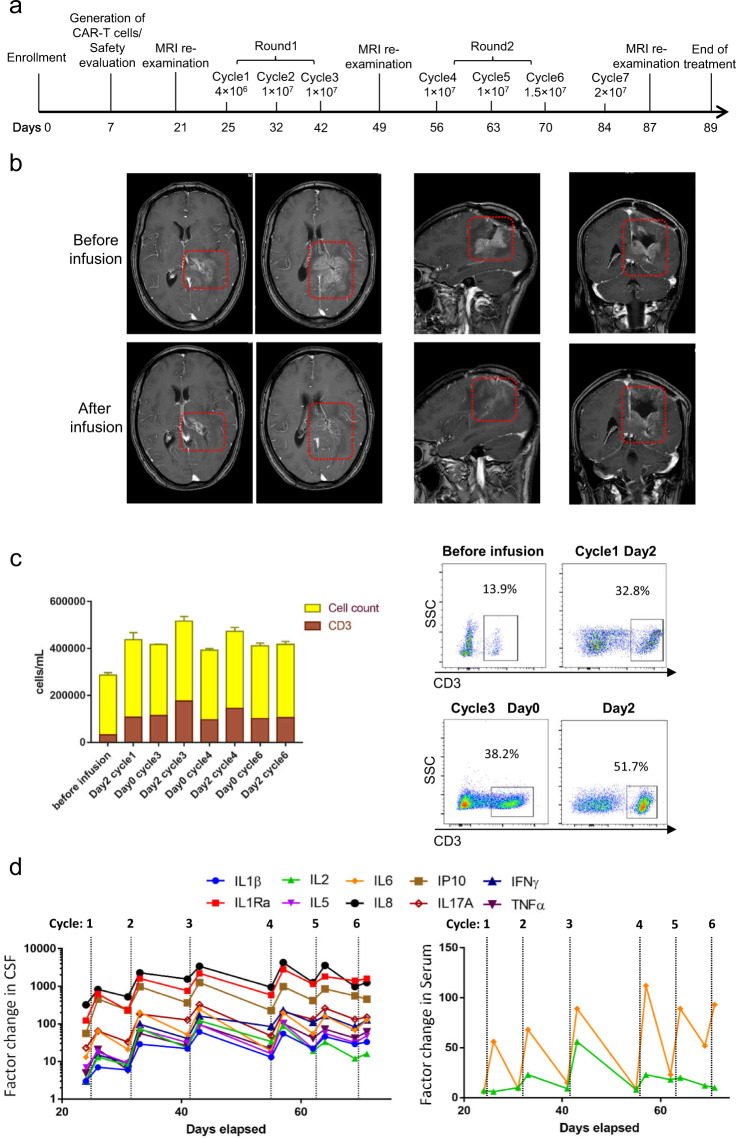
Treatment overview. **a** The schedule of two rounds of intracranial infusion of B7-H3 targeted CAR-T cells. The intracranial infusion was interrupted for 1 week after each round for assessment of safety and disease. Since the patient requested to be discharged 5 days after cycle 6 infusion and dropped out of the clinical study after cycle 7 infusion, further analysis of CAR-T therapy and resistance of post-therapy were limited. **b** Coronal, sagittal, and axial MRI of the brain before and after the first 3-cycles CAR-T cells infusion (day 21 and 49). Red dotted box highlighted the site of the resected tumor region. **c** Total nucleated cell and T cell count in the CSF, obtained from the delivery device before and after the infusion cycle 1, 3, 4, and 6. The results showed the mean values of triplicate technical repeats. Flow cytometry analysis of the T cell ratio in total nucleated cell indicated that expansion of T cells after delivery of CAR-T cell. **d** The significant cytokines changes in CSF and serum before and after each cycle infusion

Although there were no toxic effects of grade 3 or higher associated with the CAR-T cells infusion, the patient suffered from headache during cycles 1–5, which could not be completely alleviated by giving oral analgesic therapy. The headache first appeared at 3 h after cycle 1 infusion. The symptom was more obvious and repeated attack in the follow-up infusion. Treatment was paused for three days until the remission of headache in cycle 2. We thus maintained a dose of 1 × 10^7^ in the next few infusions for security. Remarkably, the lasting time of headache seemingly correlated to the infusion dose of CAR-T cells at the first-round treatment (Supplementary Table 1). In the last 3 cycles, this symptom was less obvious, in spite of higher doses of infusion (dose: 1.5 × 10^7^ and 2 × 10^7^). For evaluating the physical condition of the patient, multiple serum biochemical indexes were continuedly monitored during the treatment and results revealed no significant changes before and after local administration of the CAR-T cells (Supplementary Table 2).

After intracranial administration of CAR-T cells, evaluation of nucleated cells indicated a significant expansion of T cells in cerebrospinal fluid (CSF) samples obtained from the infusion device, especially in cycle 3 (Fig. 1c). Moreover, cell count of CSF sample collected from lumbar puncture on day 3 of cycle 3 show the existence of CAR-T cells and expansion of T cells (Supplementary Fig. 3). Further, 16 inflammatory cytokines were measured for evaluating immunologic changes in CSF and periphery blood before and after each cycle infusion. As a result, levels of 10 cytokines increased by a factor of more than 5 from pre-infusion baseline levels in CSF, and the cytokines level decreased between weekly treatment cycles. Of interest, IL2 and IL6 especially IL6 level increased significantly in periphery blood (increased by a factor of more than 5), though the extent was less obvious than that in CSF (Fig. 1d). The measured levels for 10 cytokines were provided in Supplementary Table S3.

In this subject, although B7-H3 targeted CAR-T cells mediated a short-term anti-tumor response in situ. However, the tumor became resistant to the therapy despite higher doses of CAR-T cells in later cycles. One of the possible reasons for tumor resistance was target antigen heterogeneity. Pre-therapy IHC result indicated a heterogenous expression in tumor specimen obtained before infusion. Analysis of CSF showed that the expansion of T cells was limited in the later cycles. Combined with the inflammation cytokines changes result, we supposed that CAR-T cells were not capable to eliminate all the tumor cells completely, especially B7-H3^−/low^ tumor cells. These tumor cells resisted to the therapy and relapsed. Since the patient dropped out of the clinical study, the post-therapy analysis was limited. Yet, such phenomenon of antigen heterogeneity was also detected in CAR-T therapy of GBM targeting EGFRvIII and IL13Ra2.^1,2^ The heterogeneous expression of the two target proteins were also observed in this subject (Supplementary Fig. 4). In several phase I clinical trial, a bispecific CAR, targeting CD19/CD22, was proposed to overcome antigen heterogeneity and escape in treating recurrent or refractory B cell Malignancies (NCT03241940, NCT04029038), suggesting the potential clinical benefit of targeting multi-antigen.

Another possible factor for tumor resistance was infusion dose. The clinical study maintained a relatively lower infusion dose (doses range from 4.0 × 10^6^ to 2.0 × 10^7^) in case of side effect aggravation, compared with the doses used in other clinical CAR-T therapy reports.^5^ Thus, we cannot exclude a possibility that these doses of CAR-T cells could not trigger an indirect tumor-killing effect, induced by multiply immune cells after CAR-T cells activation. This efficacy could eliminate B7-H3^−/low^ tumor cells effectively. As future studies evaluate the treatment of GBM with B7-H3 targeted CAR-T therapies it will be important to assess effectively, durable, and safe infusion doses of CAR-T cells.

Delivery route was also a key point for the CAR-T therapy against brain tumor. In this study, an Ommaya device was implanted for intracranial infusion. This device not only enabled repetitive delivery of CAR-T cells into tumor cavity through subcutaneous injection but also facilitated analyzing and monitoring the therapy. In our previously reported case of CAR-T therapy against anaplastic meningioma, the traffic of CAR-T cells was limited in the region near infusion device. But in this subject, CAR-T cells could be detected in the CSF sample collected from the lumbar puncture, indicating successful delivery of CAR-T cells into CSF circulation. The main cause of the difference was that the CAR-T cells were delivered into lateral ventricle in this subject. This observation supposed the potential trafficking of CAR T cells to distant tumor focus via lateral ventricle infusion.

One of the primary objectives of the clinical study was assessment of safety. During cycles 1–5, the patient suffered from recurrent headache. While in the last 3 cycles, this symptom was less obvious despite of higher doses of infusion. Combined with the evaluation of nucleated cells and cytokines in CSF, we supposed that the potential cause of headaches was inflammation response.

In summary, this patient was the first GBM patient enrolled in the study. Our finding supports the potential of B7-H3 targeted CAR-T therapy against GBM. Future studies will focus on tumor antigen heterogeneity and therapy-resistance mechanism.

## Supplementary information

Supplementary Materials
