# Supplementary material for: Administration of B7-H3 targeted chimeric antigen receptor-T cells induce regression of glioblastoma
Source: Signal Transduct Target Ther. 2021 Mar 26;6:125. doi: 10.1038/s41392-021-00505-7 (PMC7994554; doi:10.1038/s41392-021-00505-7)
Supplement: Supplementary file 1 — Supplementary Materials [file 41392_2021_505_MOESM1_ESM.pdf]

Supplementary Materials for

Administration of B7-H3 targeted chimeric antigen receptor-T cells induce regression of glioblastoma

Xin Tang, Yuelong Wang, Jianhan Huang, Zongliang Zhang, Fujun Liu, Jianguo Xu, Gang Guo, Wei Wang, Aiping Tong, Liangxue Zhou

Correspondence to: aipingtong@scu.edu.cn； liangxue_zhou@126.com

This PDF file includes:

Materials and Methods
Figures. S1 to S4
Tables S1 to S4

Other Supplementary Materials for this manuscript include the following:

Materials and Methods

Study approval

This phase 1 study (ChiCTR1900023435) focus on evaluating CAR-T cell therapy against central nervous system (CNS) malignant tumor. The clinical protocol was approved by the West China Hospital of Sichuan University Biomedical Ethics Committee (Ethical approval document: 2018-061). Participating patient has provided written informed consent.

Study design

Eligible patients were adults, age＞18, with histologically-confirmed diagnosis of a B7-H3$^+$ recurrent CNS malignant tumors including high grade glioma (glioblastoma), neuroblastoma and meningioma (WHO III), which were overall resistance to standard treatment including surgery and chemoradiotherapy. The minimum degree of B7-H3 histochemistry score (H-score) was 100 (0-300). The patient must also meet the following conditions including radiographic evidence of measurable disease, Karnofsky performance status (KPS) > 60, adequate cardiopulmonary function and a survival expectation＞3 months. The patient must not combine with any other types of tumor, systemic infection, or autoimmune diseases, nor require glucocorticoid treatment.

Enrolled patients were required for venous blood collection in order to harvest T cells and engineer B7-H3 targeted CAR-T cells. After evaluating CAR-T cell phenotypes, the cells were cryopreserved for later use. Enrolled patients were implanted with an Ommaya device in the location of tumor resection and underwent baseline MRI before the administration of CAR-T cells. The designed treatment program contained 12 doses of CAR-T cell via intracavitary infusion within 4 months. Clinical responses were assessed by MRI imaging after each round of infusion. Adverse events during CAR-T cell treatment were graded according to the NIH Common Terminology Criteria for Adverse Events (CTCAE), version 5.0 (http://ctep.cancer.gov).

Manufacture of B7-H3 targeted CAR-T cells

The CAR sequence was codon optimized and contains a human CD8α leader peptide, an anti-B7-H3 single chain variable fragment (scFv), a human CD8α hinge domain, a human CD8α transmembrane domain and the cytoplasmic domain of 4-1BB/CD3ζ. A truncated CD19 (CD19t) used for CAR detection was separated by a P2A ribosome skip sequence from the CAR sequence. The anti-B7-H3 scFv sequence mentioned above were derived from a highly specific monoclonal antibody (mAb) against B7-H3 (clone: mAb-J42) generated by our group using standard hybridoma technique.

For lentivirally transduced CAR-T cells manufacturing, peripheral blood mononuclear cells (PBMC) were isolated by density gradient centrifugation using Lymphoprep (Greiner Bio-One) on the day of venous blood collection. PBMC were then incubated with anti-CD25 and anti-CD45RA microbeads (Miltenyi Biotec) for negative selection to remove naïve T and Tregs (since these Tregs are known to express high levels of CD25/IL-2 receptor) according to the operation instrutions. For T cell expansion, the cells were cultured in TexMACS GMP Medium (Miltenyi Biotec) and stimulated with GMP grade OKT3 (CD3 mAb: 600ng/mL, Novoprotein) and anti-CD28 antibody (CD28 mAb: 300ng/mL, Novoprotein) with addition of recombinant human (rh) IL2 (100units/mL, PeproTech) and rh-IL15 (5ng/mL, Miltenyi Biotec) in a 37°C, 5% CO_2 environment. Two days after PBMC isolating, activated T cells were transduced with lentivirus (MOI=1) by recombinant fibronectin fragment (CH-296, Novoprotein) in the presence of rh-IL2 and rh-IL15. T cells were harvested for cryopreservation after culturing 8-10 days. For cryopreservation, CAR-T cells were harvested, washed in phosphate buffer solution (PBS)

containing 2% HSA, and resuspended in Serum-Free Cell Freezing Medium (BioLife Solutions). The whole CAR-T cell manufacturing process was completed within about 2 weeks. Quality control result of the manufactured B7-H3 targeted CAR-T cells were shown supplementary Table 4.

Infusion process of CAR-T cells

Participant was pre-medicated with acetaminophen P.O. (per os) and diphenhydramine I.V. (intravenous injection) or P.O. 30 minutes before CAR-T cell infusion according to a protocol of glioblastoma CAR-T cell therapy [1]. CAR-T cell infusions were administered manually via Ommaya device in a 1.0 mL volume over approximately 10 minutes using a 21-gauge butterfly needle, followed by a 1.0 mL normal saline flush over 5 minutes. After CAR-T cell infusion, research participants were required for being monitored for 3 hours.

Clinical Imaging

MRI scans of the cerebral post-gadolinium T1-weighted sequences were acquired on a 3.0 T clinical MRI instrument (Siemens Trio Tim). The CT scans of the Ommaya device were acquired on a Philips Brilliance 16 row helical scanner. Regions of contrast-enhancing recurrent tumor and Ommaya device were confirmed by a radiologist. All the scans were analyzed on a Siemens Syngo imaging system.

Immunohistochemistry analysis

The resected tumor specimens were collected from the Neurosurgery Department of West China Hospital. For all IHC analysis, the samples were performed on a pathological section of paraffin-embedded specimens. The tumor regions were confirmed by a clinical neuropathologist. All of samples were dealt with 10% formalin and embedded by paraffin, dried for 90 minutes at 65℃, then blocked with distilled water containing 3% H_2O_2 and PBS containing 10% normal goat serum (Boster) at room temperature in turn. Slices were stained with corresponding mono-antibody at 4℃ overnight. For B7-H3 expression analysis, anti-B7-H3 mAb (clone: D9M2L, Cell Signaling Technology, CST) was used to calculate for defined tumor areas by DAB detection system (ZSGB-Bio).

Flow cytometry analysis

For all flow cytometry analysis, cells were washed twice with PBS containing 0.5% bovine serum albumin (BSA) and incubated with corresponding antibodies for 30 minutes at 4℃ in the dark. BD LSRFortessaTM (BD Biosciences) instrument was used to analyze the cells and percentages of positive cells were calculated via FlowJo-V10 software for analysis. For the analysis of CSF, all samples were stored and transported at cryogenic temperatures, then processed with ACK buffer (Sigma) and stained with the corresponding antibody. For CAR-T cell detection shown in Figure S3, anti-CD3 and anti-CD19 specific fluorescein-conjugated antibody (Biolegend) was used. The total cell count was measured by Counter-Star instrument after lysis of red blood cells using ACK buffer.

Multiple cytokines detection

Peripheral blood samples were collected in tube without EDTA and CSF samples were obtained from the reservoir of Ommaya delivery device. All samples were centrifuged at 3000 g for 10 minutes, and the supernatants were collected and frozen at −80°C. Cytokines profile was measured

using a MILLIPLEX multiplex immunoassay (Millipore) and analyzed with a Luminex FlexMap 3D system (Luminex Corporation).

CAR expression analysis by quantitative real-time reverse transcriptase polymerase chain reaction (RT-PCR)

Total RNA was prepared from the nucleated cell obtained from CSF using an Eastep® Super Total RNA Extraction kit (Promega). The purity was confirmed with an A260/A280 ratio greater than 2.0. The complementary DNA (cDNA) was synthesized using a GoScript™ Reverse Transcription Mix (Promega). RT-PCR was performed by using the Bio-Rad CFX96™ Real-Time PCR system and SsoFast EvaGreen® Supermix Mix (Bio-Rad). The reaction solution consisted of 2 μL of the template, 10 μL of the Supermix Mix (Bio-Rad), 1 μL of upstream/downstream primer (10 μM), and 6 μL of distilled water. The PCR conditions used were Enzyme activation for 30 sec at 95°C, followed by 40 PCR cycles of denaturation at 95°C for 5 s, annealing/extension at 58°C for 5 sec. CAR expression levels were normalized to that of β-actin in each sample using the ΔΔCT method.

Real-Time Monitoring of Cytotoxicity (RTCA)

The cytotoxic ability of B7-H3 targeted CAR-T cells was determined using the xCELLigence® real-time cells analyzer (ACEA Bioscience, Inc. xCELLigence RTCA SP). To start the real-time cell analysis, background readings was obtained from 100 μL of media added in each well of the E-plate® 96. In order for cell attachemnt, the primary tumor cells (10^4 cells per well) were cultured in E-plate 96 (ACEA Bioscience) for about 15 hours before CAR-T cells were added (E:T=5:1). Three replicates were available for each well. Cell index measurements were performed at 15 min intervals for 72 h. The data were acquired and analyzed using the manufacturers protocols (ACEA Bioscience, Inc. RTCA Software 2.1). The expression levels of CAR were normalized to that of β-Actin in each sample using the ΔΔCT method. The sequences of the primers used to detect β-Actin mRNA and CAR mRNA were as follows:

β-Actin forward primer (5'-GGACCTGACTGACTACCTCAT-3'),
β-Actin reverse primer (5'-CGTAGCACAGCTTCTCCTTAAT-3'),
CAR forward primer(5'- GAAGCCTCTGGATTCACTTT-3'),
CAR reverse primer(5'- TAACCATCCCAATGTCTTGC -3').

Enzyme-linked immunosorbent assay (ELISA)

CAR-T cells (1×10^5) were co-cultured with primary tumor cells (5×10^4) in 48-well plates without addition of exogenous cytokines. 12 hours after coculture, supernatants were collected, and cytokines (IFNγ and IL2) were measured using corresponding ELISA kits (Thermo-Fisher Scientific) following manufacturer's instructions. In this assay, student's t test was used for single comparisons. The experiments were repeated twice. A *P* value less than 0.05 was considered significant.

Reference

1. Brown CE, Alizadeh D, Starr R et al. Regression of Glioblastoma after Chimeric Antigen Receptor T-Cell Therapy. N Engl J Med 2016; 375: 2561-2569.

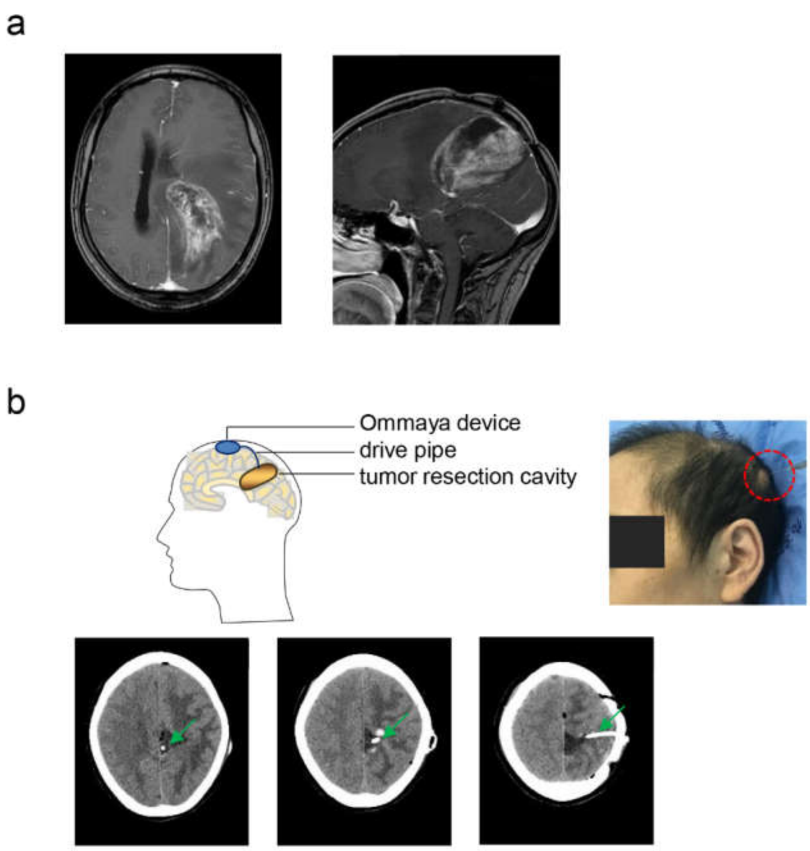

Figure. S1.

(a)the MRI images of tumor before second surgical resection. (b) Pattern diagram and CT images (the green arrow pointing at) of Ommaya device and subcutaneous location for CAR-T cell infusion (Red dotted circle).

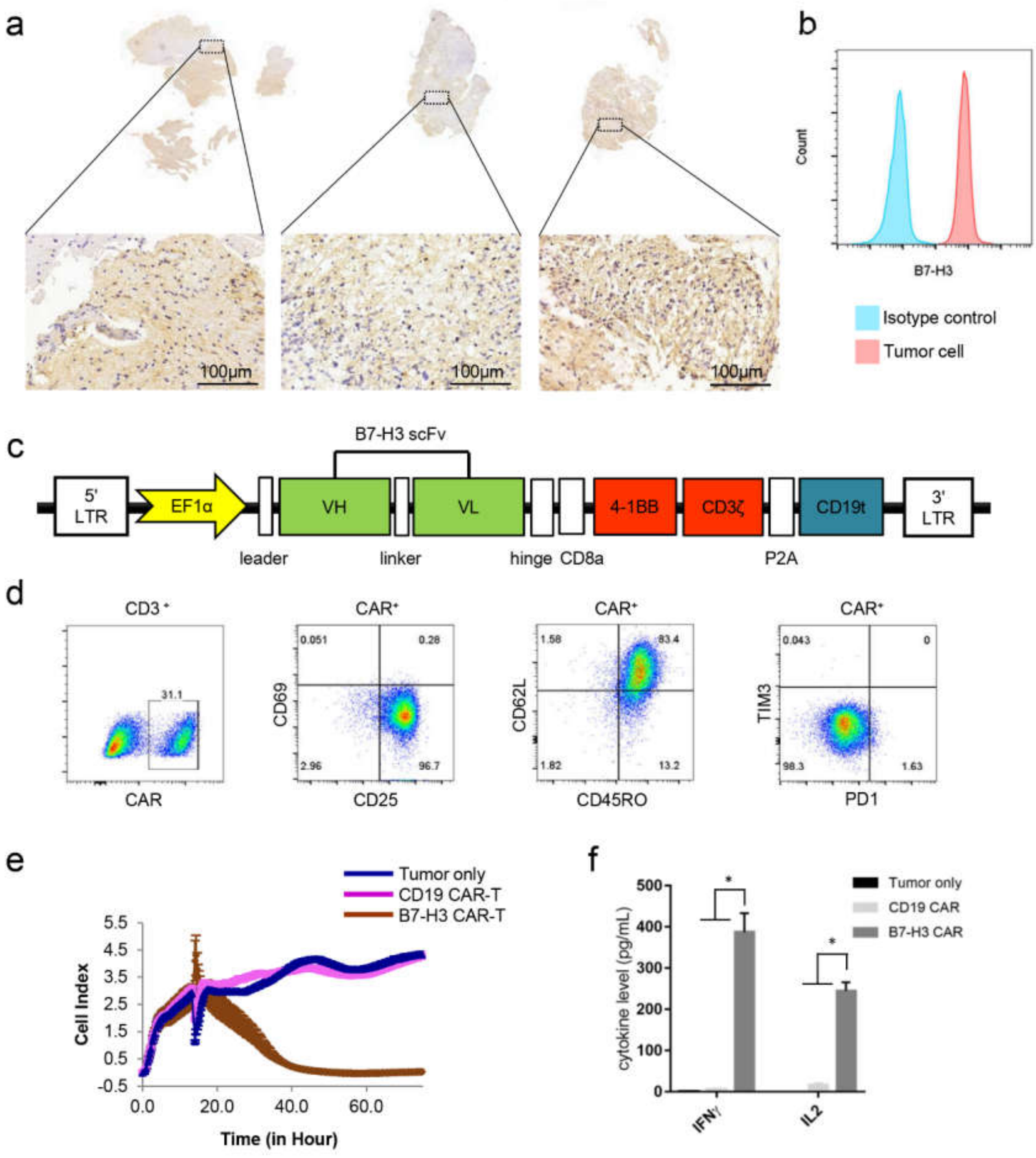

Figure. S2.

General information of the enrolled patient. (a) IHC result of primary tumor specimen obtained from the neurosurgery resection, with a histochemistry score evaluated as 110. (b) Flow cytometry result of the tumor-isolated tumor cells. Primary tumor after 3 times of passages was used for B7-H3 detection. The isotype control was used as negative control. (c) The diagram of CAR components consists of the B7-H3 targeted CAR (containing the CD8α signal peptide, B7-H3-specific scFv (derived from mAb-J42), hinge, CD8 transmembrane domain (CD8α), and the 4-1BB and CD3ζ cytoplasmic signaling domains), as well as the P2A ribosomal skip and truncated CD19 (CD19t) sequences. The opening reading frame is driven by the human EF1α promoter. (d) Cell phenotype of the manufactured B7-H3 targeted CAR-T cells. (e) Real-time

monitoring of cytotoxicity assay of CAR-T cells against primary tumor cells. (f) Cytokine (interferon γ and IL2) secretion levels were measured to analyze immunological changes in B7-H3/CD19-targeted CAR-T cells cocultured with primary tumor cells (40,000 T cells to 10,000 tumor cells) for 12 h by ELISA. Though the total cytokines level were relatively low, the results were still statistically significant. *$P < 0.05$. For RTCA and ELISA, experiment technical repeats were done in triplicates.

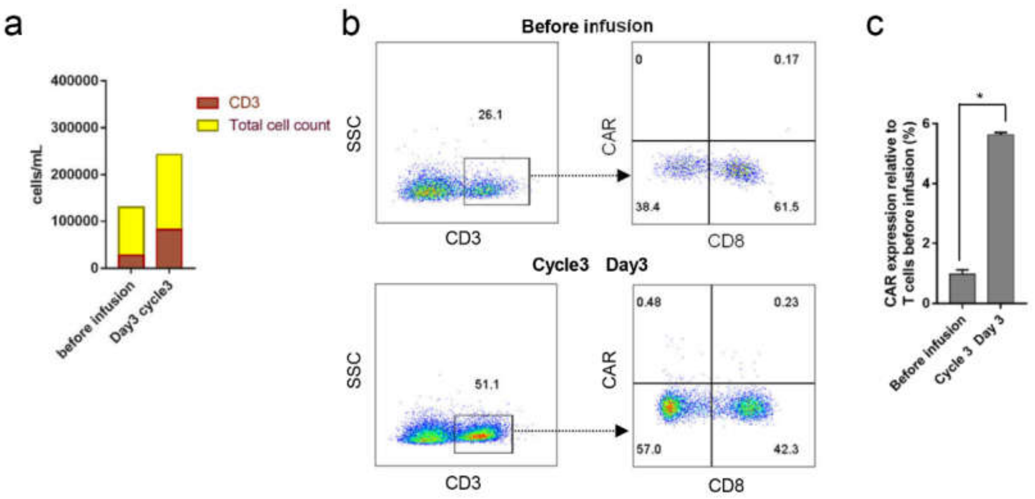

Figure. S3.

Detection of B7-H3 targeted CAR-T cell in CSF, collected from the lumbar puncture operation. (a) Total nucleated cell and T cell count in the CSF, obtained before and after the infusion cycle 3. (b)Flow cytometry analysis of the T cell ratio in total nucleated cell indicated that expansion of T cells after delivery of CAR-T cell. For CAR staining in flow cytometry, an anti-CD19 antibody was used. (c) CAR expression was analyzed by quantitative real-time reverse transcriptase polymerase chain reaction. *P < 0.05. For RT-PCR, experiment technical repeats were done in triplicates.

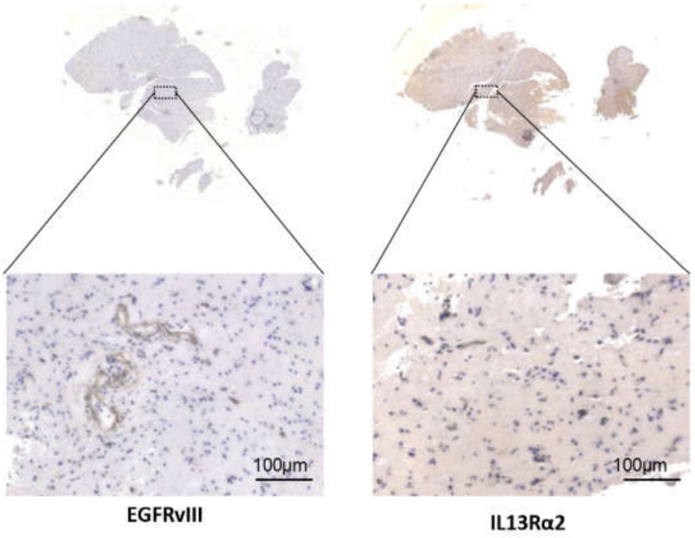

Figure. S4.
EGFRvIII and IL13Rα2 staining IHC result of primary tumor specimen obtained from the neurosurgery resection.

Table S1.

Records of adverse events

Cycle	CAR-T cell doses	Adverse event	Lasting (Days)	Worst grade
1	4×10^{6}	Headache	4	I
2	1×10^{7}	Headache	7	II
		Dizziness	3	I
3	1×10^{7}	Headache	7	II
4	1×10^{7}	Headache	5	II
5	1×10^{7}	Headache	3	I

Grade of adverse events based on NIH Common Terminology Criteria for Adverse Events (CTCAE), version 5.0 (http://ctep.cancer.gov).

Table S2.

Serum biochemical index during CAR-T cell Therapy.

Items	D25	D26	D32	D33	D42	D43	D56	D57	D63	D64	D70	D71	D80	Units	Reference value
Alanine aminotransferase (ALT)	9	12	7	6	5	5	7	8	5	5	8	6	6	IU/L	7-40
Aspartate aminotransferase (AST)	13	14	10	12	8	8	9	13	9	13	11	10	11	IU/L	13-35
Glutamyltranspeptidase (GGT)	12	13	13	15	12	13	12	15	14	12	15	14	9	IU/L	7-45
Total bilirubin (TBIL)	16.7	16.7	12.5	20.6	9	9.8	11.2	10.3	12.9	14.7	11.7	12.1	11.3	μmol/L	5.0-28.0
Direct bilirubin (DBIL)	5.2	5.7	4.2	6.3	2.9	3.6	5.1	4.5	5.2	5.6	5.5	5.4	6.3	μmol/L	＜8.8
Indirect bilirubin (IBIL)	11.5	10.4	8.3	14.3	6.1	6.2	6.1	5.8	7.7	9.1	6.2	6.7	5	μmol/L	＜20
Carbamide (BUN)	3.1	2.6	2.4	2	2.3	2.1	2.2	2.9	3.4	4	4.1	3.4	2.2	mmol/L	2.9-7.7
Creatinine (CREA)	67	38	51	50	53	55	39	48	43	45	43	38	41	μmol/L	37-110
Serum cystatin C (Cys-C)	0.83	0.6	0.95	0.82	0.57	0.47	0.55	0.49	0.56	0.68	0.65	0.63	0.51	mg/L	0.51-1.09
Trioxypurine (URIC)	208	164	152	130	180	171	165	158	166	191	159	151	152	μmol/L	160-380
Estimated glomerular filtration rate (eGFR)	88.26	114	103	104	102	101	103	105	109	107	109	114	102	ml/min/L	56-122
Sodium (Na)	144	141	142	137	141	144	143	143	140	140	140	144	140	mmol/L	137-147
Potassium (K)	4.13	3.94	4.15	3.68	4.57	3.62	3.29	3.48	3.56	3.93	3.31	3.1	3.61	mmol/L	3.5-5.3
Chloride (Cl)	104	101	103	100	101	103	101	99	102	101	98	101	96	mmol/L	99-110
Calcium (Ca)	2.3	2.19	2.35	2.25	2.2	2.22	2.24	2.24	2.3	2.25	2.23	2.34	2.26	mmol/L	2.1-2.7

Table S3.

CSF and Serum cytokines level before and after CAR-T cell infusion

sample	cytokine	D24	D26	D31	D33	D41	D43
CSF	IL1Ra	123.26	630.15	230.54	1620.39	761.48	2205.06
	IL1β	*3.1	7.12	6.34	29.78	22.75	62.45
	IL2	3.11	13.52	8.4	75.35	23.4	12.65
	IL5	7.21	18.52	9.45	72.53	32.27	96.34
	IL6	13.57	103.7	20.11	263.12	52.45	343.27
	IL8	322.54	826.8	526.3	2305.78	1560.41	3438.06
	IP10	56.77	458.65	231.28	972.64	365.94	1260.6
	IL17A	23.1	62.45	33.84	182.25	127.57	326.34
	TNFα	5.61	21.78	6.14	56.42	26.17	96.37
	IFNγ	3.23	15.22	9.63	97.51	41.41	162.3
peripheral blood	IL1Ra	201.33	261.61	268.13	288.79	332.67	541.51
	IL1β	OOR <	OOR <	*1.06	*1.22	*2.27	*3.93
	IL2	4.32	3.28	10.5	23.41	9.14	56.22
	IL5	*4.93	*5.25	*6.23	*5.36	13.00	9.46
	IL6	6.16	56.4	9.1	68.24	15.34	84.88
	IL8	39.62	56.68	59.3	68.91	62.58	113.84
	IP10	233.45	312.76	335.55	298.54	346.98	366.78
	IL17A	18.03	19.86	20.63	22.48	19.79	18.31
	TNFα	11.67	13.68	13.95	20.12	22.63	32.44
	IFNγ	2.19	3.66	4.12	5.23	7.86	8.33

sample	cytokine	D55	D57	D62	D64	D69	D71
CSF	IL1Ra	585.43	2830.45	1150.02	1830.22	1405.54	1605.38
	IL1β	13.13	55.97	22.34	46.78	29.24	33.98
	IL2	34.12	89.78	19.5	33.88	12.56	16.04
	IL5	16.72	106.63	22.47	56.36	32.67	48.32
	IL6	19.96	218.6	55.93	162.32	68.97	126.45
	IL8	951.06	4320.71	1260.32	3620.44	980.24	1260.32
	IP10	226.45	982.66	421.11	855.37	562.79	456.9
	IL17A	48.6	212.75	132.48	263.45	132.94	152.61
	TNFα	22.82	103.89	42.36	73.77	42.35	62.67
	IFNγ	85.32	226.58	108.76	166.24	83.64	126.4
peripheral blood	IL1Ra	410.22	499.13	513.34	450.7	422.69	433.19
	IL1β	*2.46	*3.89	*4.03	*4.32	*3.29	*2.61
	IL2	8.14	23.45	18.92	20.78	12.4	10.2
	IL5	8.23	9.88	11.07	10.36	9.22	9.87
	IL6	9.1	92.41	23.87	79.65	33.35	41.57
	IL8	102.15	132.49	125.52	120.68	113.34	129.63
	IP10	265.85	299.64	213.49	200.13	198.64	233.18
	IL17A	16.23	15.49	17.89	18.37	15.66	16.32
	TNFα	20.35	25.48	22.75	19.24	18.7	16.52
	IFNγ	5.01	6.35	4.95	3.68	4.64	5.83

Cytokine unit: pg·mL^{-1}
OOR<: The index signal value is below the standard curve detection range.
*: The index signal value is lower than the minimum standard concentration, but the corresponding concentration can be calculated by multi-parameter standard curve.

Table S4.

Quality control of B7-H3 targeted CAR-T cell

Test	Specification	Result
Endotoxin	＜10EU/mL	2.1-3.8 EU/mL
Mycoplasma	Negative	Negative
Sterility assay	Negative	Negative
In vitro viral assay	Negative	Negative
Cell viability	＞70%	93%
CAR expression	report result	30-40%
Surface phenotype	report result	$CD3^{+}$, $TCR\alpha\beta^{+}$ (including $CD4^{+}$ and $CD8^{+}$)
B7-H3 specific cytolytic activity	report result	＞20% specific lysis at E:T of 5:1
